# Supplementary material for: Mesenchymal stromal cell-derived septoclasts resorb cartilage during developmental ossification and fracture healing
Source: Nat Commun. 2022 Jan 28;13:571. doi: 10.1038/s41467-022-28142-w (PMC8799643; doi:10.1038/s41467-022-28142-w)
Supplement: Supplementary file 1 — Supplementary information [file 41467_2022_28142_MOESM1_ESM.pdf]

Supplementary information:

Mesenchymal stromal cell-derived septoclasts resorb cartilage during developmental ossification and fracture healing

Kishor K Sivaraj[1], Paul-Georg Majev[1], Hyun-Woo Jeong[1], Backialakshmi Dharmalingam[1], Dagmar Zeuschner[2], Silke Schröder[1], M. Gabriele Bixel[1], Melanie Timmen[3], Richard Stange[3], and Ralf H. Adams[1#]

[#]Corresponding author : Ralf H. Adams

Supplementary Figures

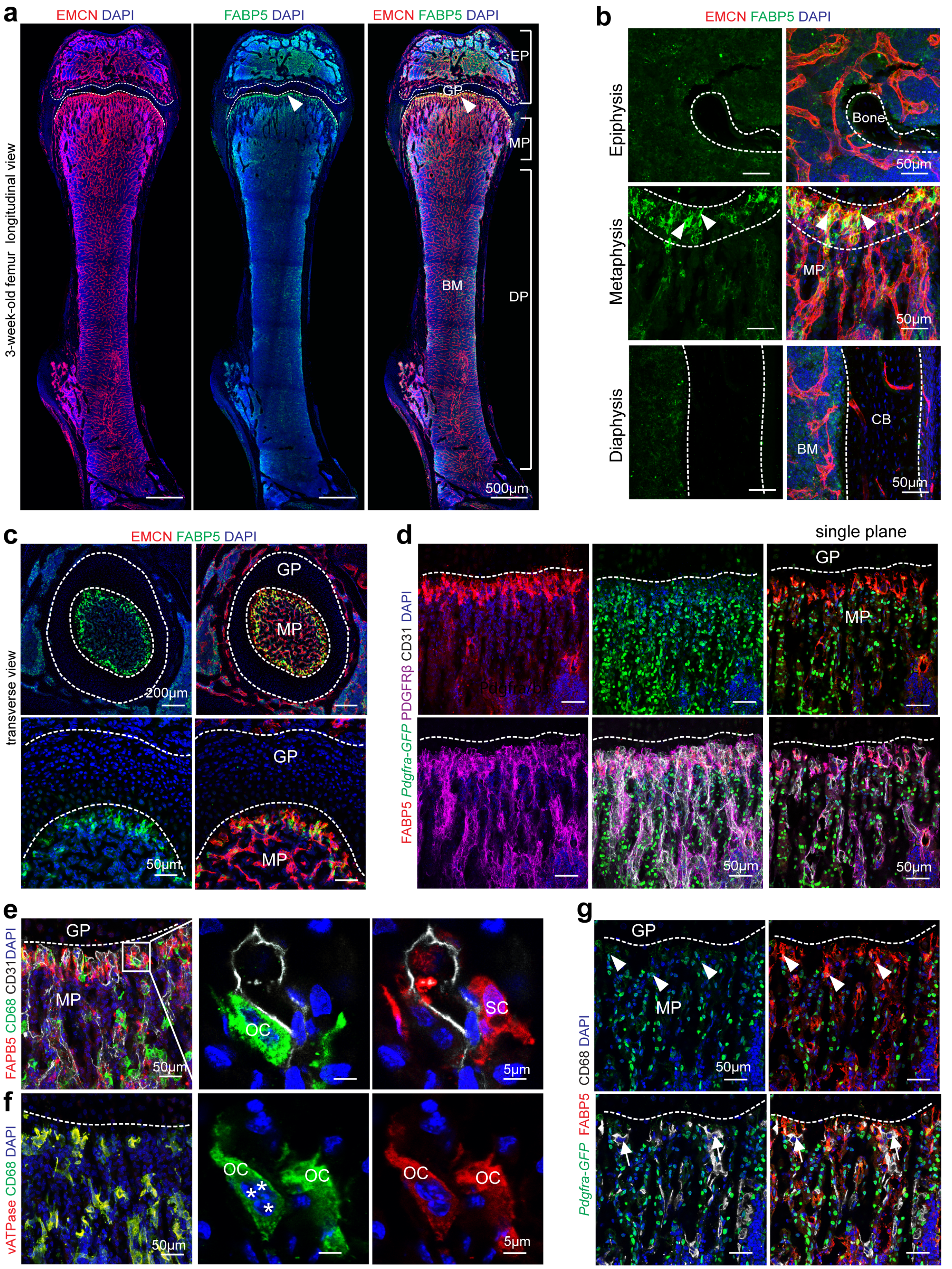

Supplementary Figure 1. Localization and features of SCs. a and **b**. Tile scan confocal images of longitudinal sections of 3-week-old wild-type femoral metaphysis. FABP5+ (green,

arrowhead) SCs are associated with distal EMCN+ (red) bud ECs. DAPI (blue) (**a**). Representative confocal images show different region of femur, epiphysis (EP), metaphysis (MP), and diaphysis (DP). FABP5+ (green) SCs are mainly present at the chondro-osseous border near growth plate (GP) (**b**). Bone marrow (BM), cortical bone (CB). **c**. Representative confocal images of transverse sections of epiphysis showing metaphyseal vessels (EMCN, red) associated with SCs (FABP5, green). **d**. 3-week-old *Pdgfra-GFP* metaphysis with GFP signal and PDGFRβ immunostaining (magenta) in FABP5+ (red) SCs near growth plate (GP). ECs, CD31 (gray). DAPI in blue. Overview image for Figure 1c. **e** and **f**. Confocal images showing FABP5+ (red) SCs close to CD68+ (green) OCs and CD31+ ECs (gray) (**e**). OCs contain multiple DAPI-stained (blue) nuclei (asterisks), and express vATPase (red) and CD68 (green) (**f**). **g.** Representative confocal images of 3-week-old *Pdgfra-GFP* metaphysis. SCs (FABP5, red) are GFP+ (green; arrowheads), whereas CD68+ osteoclasts (gray) lack GFP signal (arrow). DAPI in blue. Supplementary figure 1a to g (n =5-6) independent biological samples.

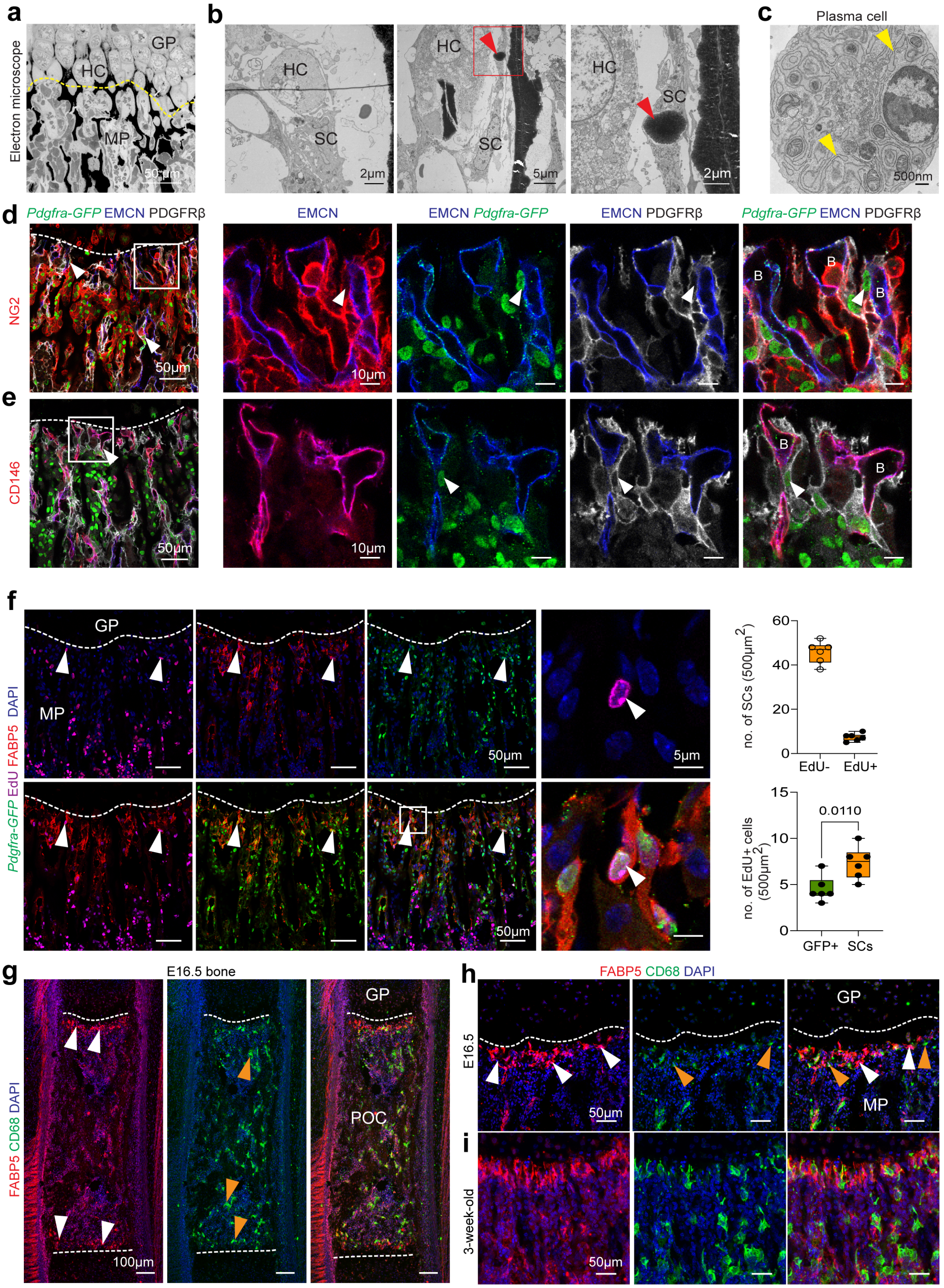

Supplementary Figure 2. Characterization of septoclasts. a and **b**. Electron micrographs of metaphysis region. SCs are located close to ECs and hypertrophic chondrocytes (HC) in the metaphysis (MP) (**a**). SCs physically interact with chondrocytes and contain electron dense

particles (right image) (**b**). **c**. Electron micrograph image of plasma cell from murine spleen with enlarged ER. **d** and **e**, Confocal images showing expression of NG2 (**d**) and CD146 (**e**) in 3-week-old *Pdgfra-GFP* femur. NG2 (red) marks bone cells, BMSCs and SCs. Signal partially overlaps with *Pdgfra-GFP* (green) and PDGFRβ (white) immunostaining (arrowheads) (**d**). CD146 is mainly expressed by metaphyseal endothelial cells (arrowheads) (**e**).

f. Representative confocal images showing EdU+ (magenta) FABP5+ (red) proliferating SCs (arrowheads) near vessel buds (B) in 3-week-old *Pdgfra-GFP* (green) femur. Images on the right show higher magnifications of boxed area. Upper graph on the right shows numbers of non-proliferating (EdU-) and proliferating (EdU+) FABP5+ SCs. The number of EdU+ metaphyseal GFP+ FABP5- and GFP+ FABP5+ cells is shown at the bottom (n=6 independent biological samples; data are presented as mean values +/- SEM, Statistical analysis performed using two-tailed Mann-Whitney test). Source data are provided in Source Data file. **g** and **h.** Confocal image of E16.5 bone sections showing CD68+ (green; orange arrowheads) OCs throughout the primary ossification centre (POC) (**g**), whereas FABP5+ (red) SCs (white arrowheads) are concentrated at the vascular front near growth plate (GP). **i.** Confocal images of 3-week-old bone show that FABP5+ SC are highly concentrated near the growth plate compared to CD68+ OCs. Supplementary figure 2a to c (n =3) and d, e, g-i (n =4) independent biological samples.

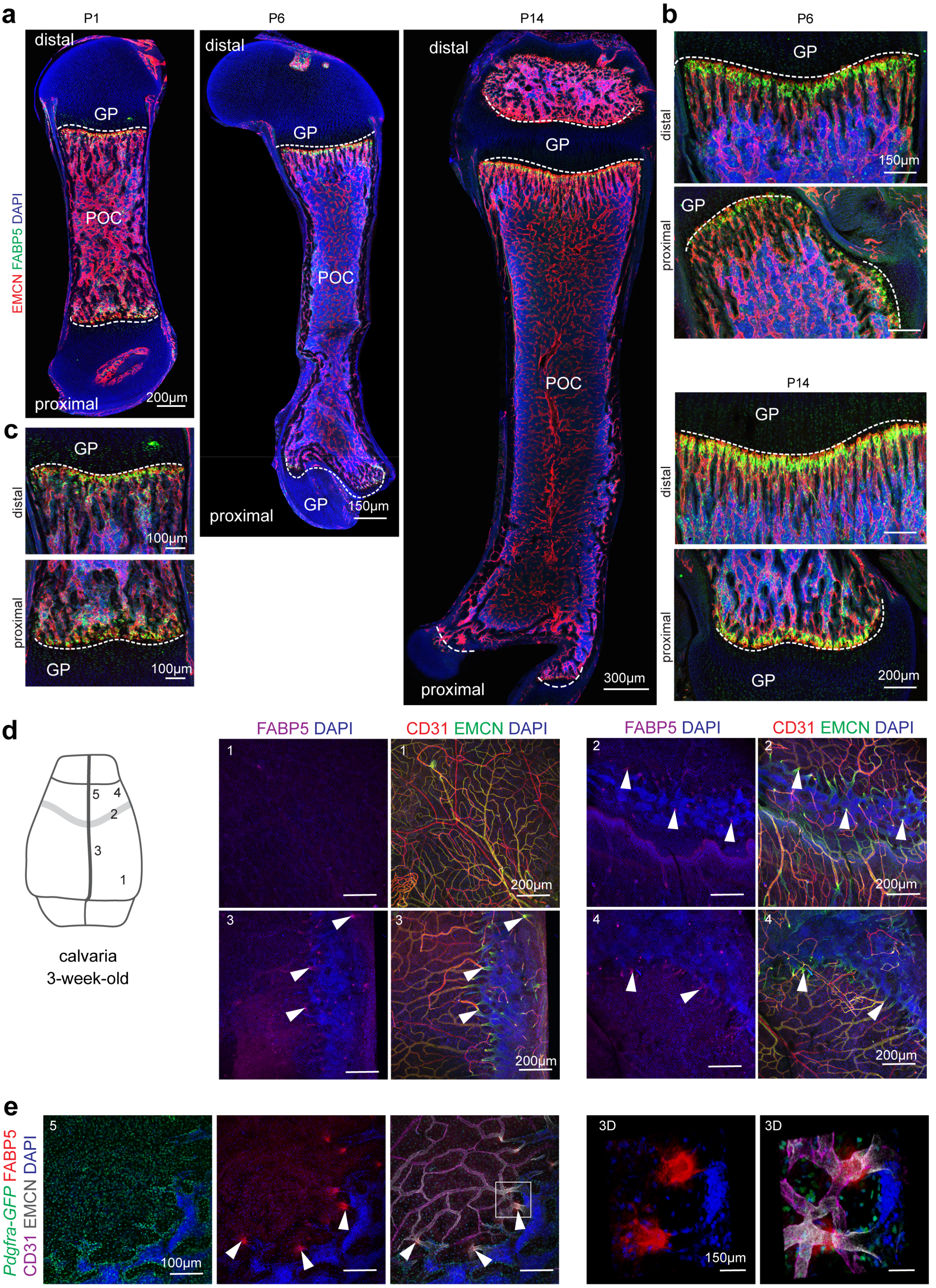

Supplementary Figure 3. Distribution of FABP5+ cells in long and flat bone. a to **c**. Tile scan confocal longitudinal image of femur at postnatal day 1 (P1), P6 and P14. SCs (FABP5,

green), ECs (EMCN, red) and nuclei (DAPI, blue) are stained (**a**). High magnifications of distal and proximal metaphysis with vessel-associated SCs near growth plate (GP, dashed lines) (**b**, **c**). **d**. FABP5+ cells in flat bone. Confocal images of different regions of 3-weeks-old calvaria show absence of cells with expression of FABP5 (magenta). Sparse cells with low FABP5 expression (arrowheads) are seen in association with CD31+ (red) or EMCN+ (green) vessels. Nuclei, DAPI (blue). **e**. Representative confocal image of 3-week-old *Pdgfra-GFP* calvaria with FABP5+ (red, arrowhead) cells. Higher magnification 3D images shown on the right. Supplementary figure 3a to e (n =4) independent biological samples.

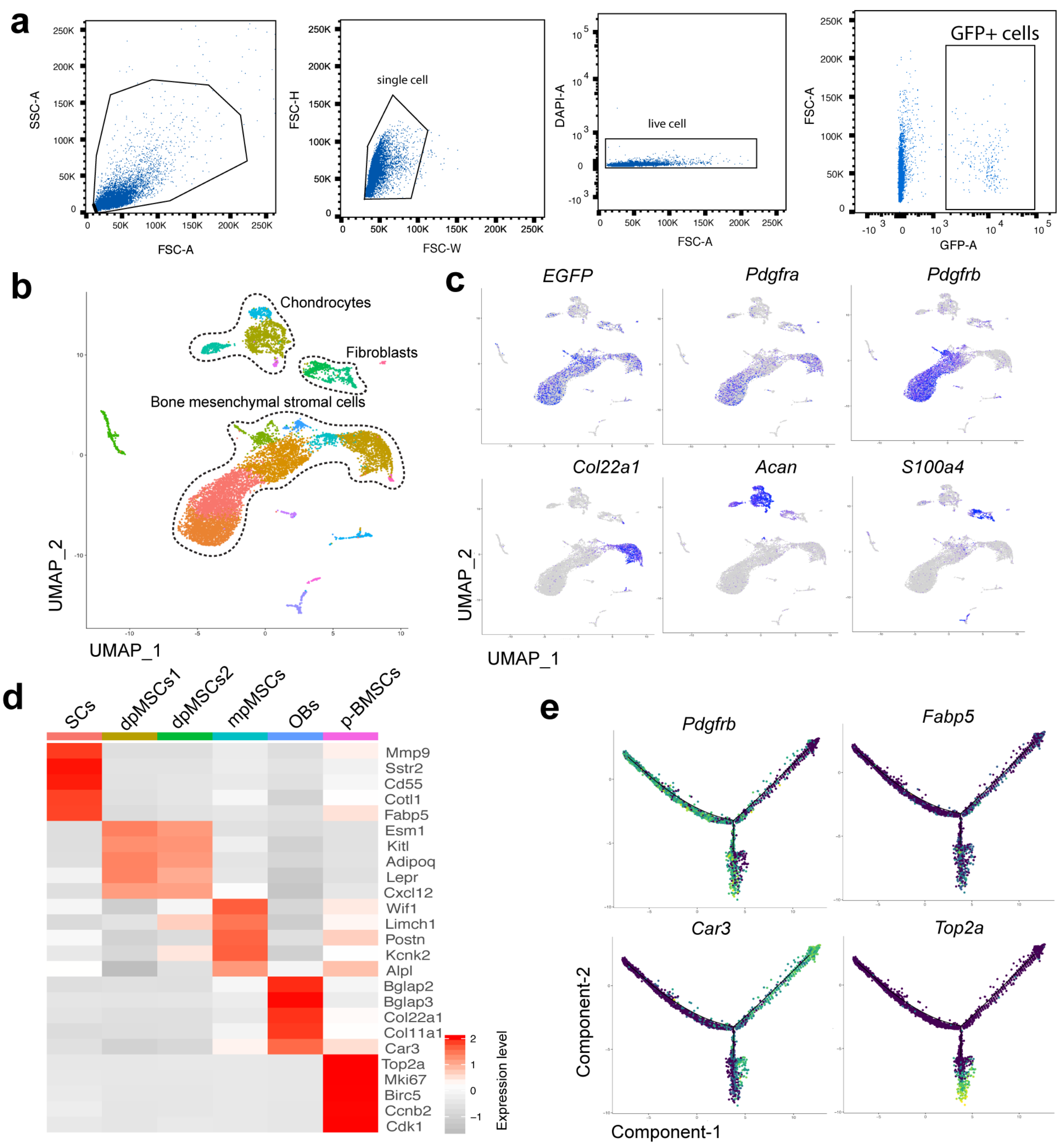

Supplementary Figure 4. scRNA sequencing of septoclasts. a. Representative gating strategy for the FACS isolation of GFP+ cells from 3-week-old *Pdgfra-GFP* metaphysis and epiphysis for scRNA-sequencing. **b** and **c.** UMAP plot of *Pdgfra-GFP* scRNA-seq data with colour-coded cell clusters (**b**). *Pdgfra-GFP* expression and cell type-specific marker genes are displayed in feature plots (**c**). **d**. Subclustering of non-haematopoietic bone-derived cells based on highly expressing 5 marker genes shown in heatmap. **e.** Monocle trajectory analysis of bone mesenchymal stromal cell differentiation. Relative expression of the indicated genes displayed in pseudo-time.

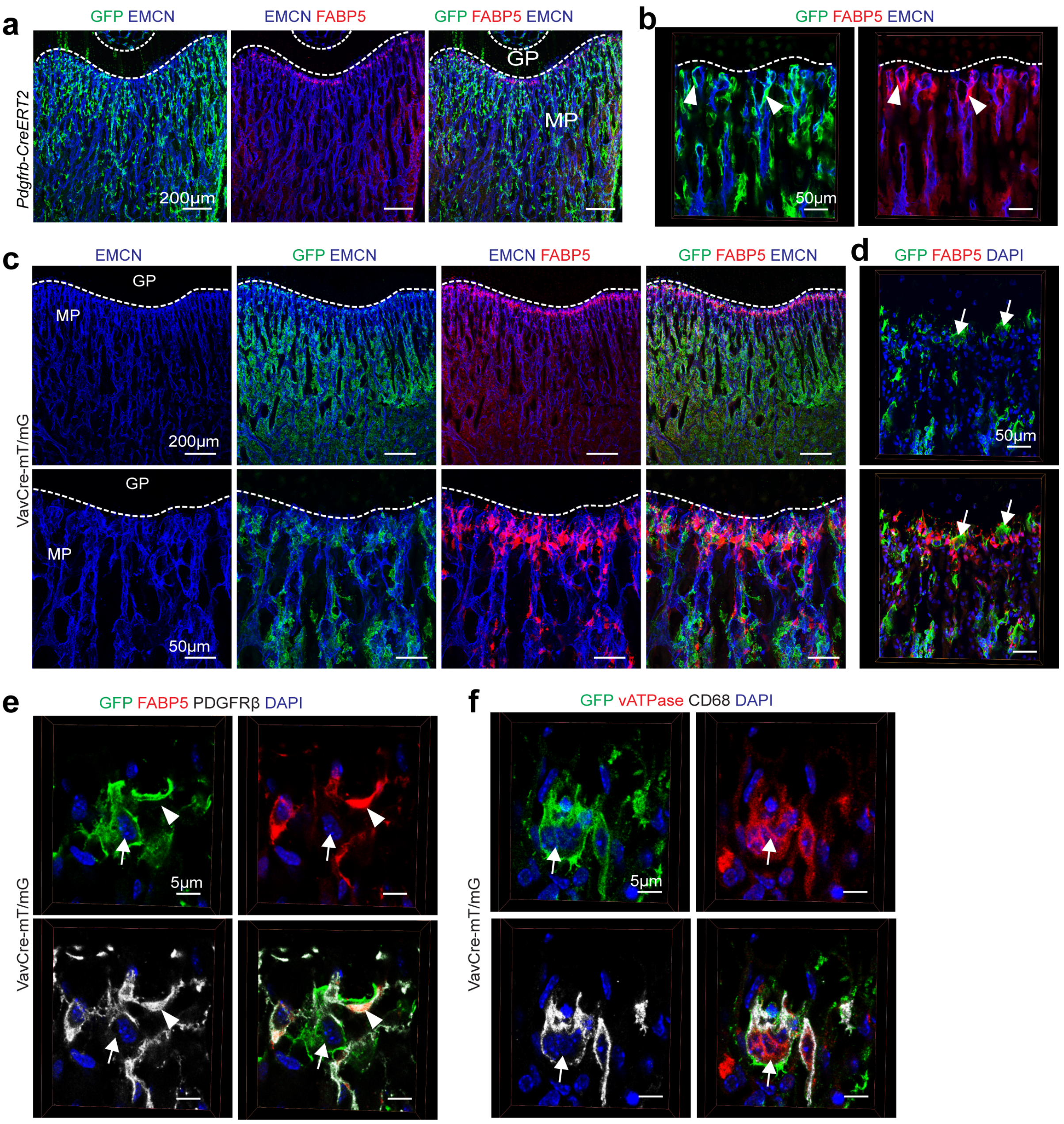

Supplementary Figure 5. Genetic lineage tracing of septoclasts. a and **b**. Representative confocal images for lineage tracing with inducible *Pdgfrb-CreERT2 R26-mTmG* mice after tamoxifen treatment from P1-3 and analysis at P21. FABP5+ SCs show GFP labelling (**a**). High magnification images (**b**). **c** and **d**. Haematopoietic cell labelling in *Vav1-Cre R26-mTmG* mice. GFP (green) expression does not mark FABP5+ SCs (red, arrowheads) (**c**), whereas osteoclasts are GFP+ (arrow) but FABP5- (**d**). **e** and **f**, Confocal high magnification and single plane images showing *Vav1-Cre*-labelled GFP+ cells lack PDGFRβ (gray, arrowhead) or FABP5 (red) immunostaining (**e**) but express the osteoclast markers vATPase (red) and CD68 (gray) (**f**). Nuclei, DAPI (blue). Supplementary figure 4a to f (n =4) independent biological samples.

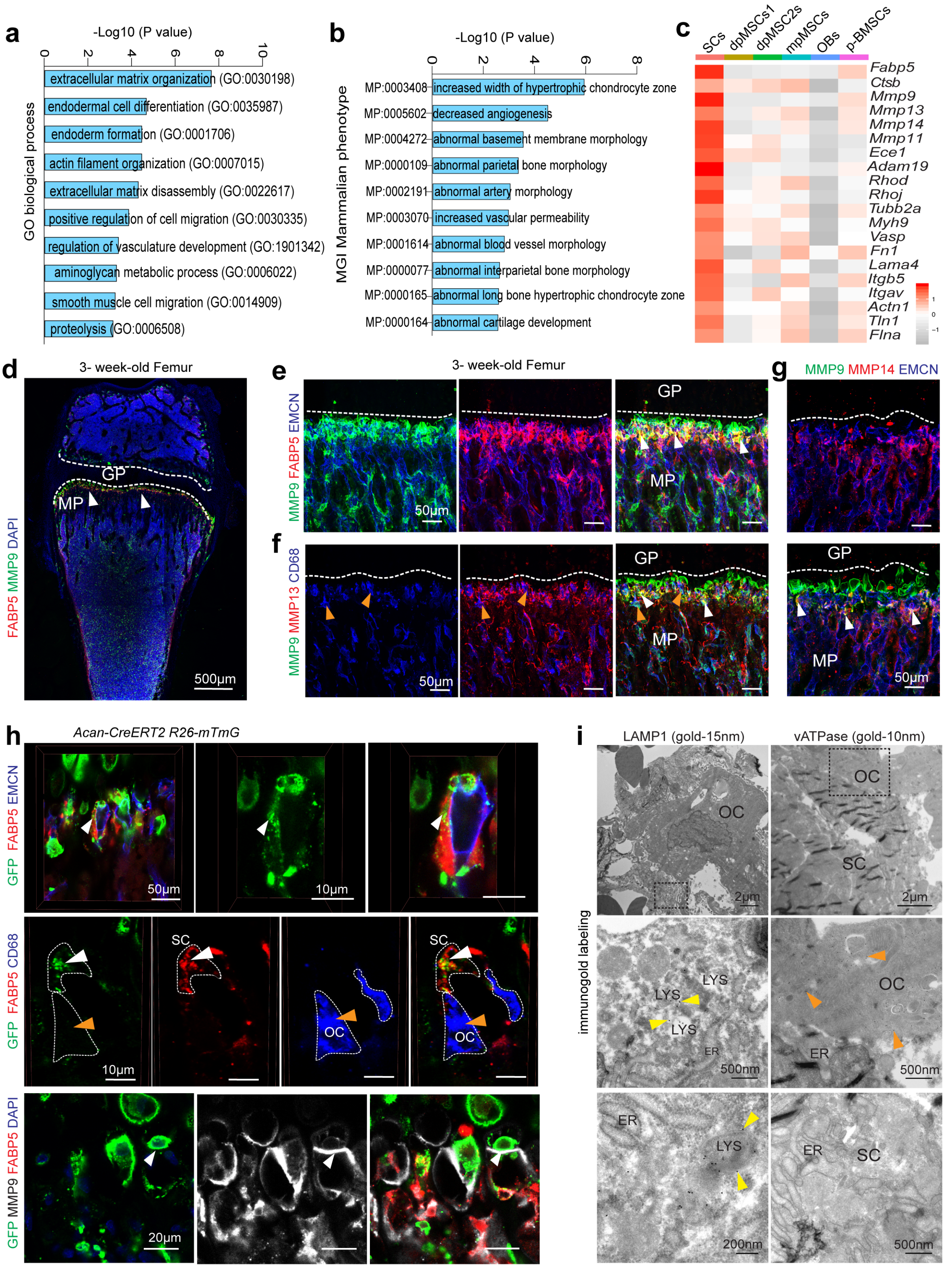

Supplementary Figure 6. Septoclast function and MMP expression in bone. a and **b**. Gene-set enrichment analysis of upregulated genes in SCs. Gene ontology (GO) analysis listing enriched biological processes (**a**). Association of SC-enriched genes with MGI mammalian

phenotypes (**b**). Size of the bar graph represents significance (p-value). **c**. Heatmap of highly upregulated genes in SCs. **d** to **e**. Tile scan confocal overview images of 3-week-old wild-type femur and higher magnification of metaphysis (right panels) showing high MMP9 (green) staining and FABP5+ (red) SCs at the chondro-osseous interface facing the growth plate (GP) (**d**). Higher magnifications show MMP9 (green) immunosignals in FABP5+ (red) SCs (arrowheads) and potentially secreted MMP9 in proximity of growth plate (GP) (**e**).

f and **g**. Confocal images showing enrichment of MMP13 (red) and MMP9 (green) expression (white arrowheads) in proximity of growth plate (GP, dashed line). Lower MMP9 signals mark CD68+ (blue) OCs (orange arrowheads) (**f**). MMP14 (MT-MMP1, red, arrowheads) signal partially overlaps with high MMP9 (green) staining (**g**). According to scRNA-seq data, SCs express MMP9, MMP13 and MMP14. **h.** *Acan-CreERT2*-controlled recombination of the *R26-mTmG* reporter labels chondrocytes expressing the cartilage-specific proteoglycan core protein (Aggrecan). FABP5+ (red) SCs are not targeted by *Acan-CreERT2.* GFP+ chondrocytes fragments (arrowheads in top and middle row) inside FABP5+ (red) SCs but not CD68+ (blue) osteoclasts (OC, orange arrowheads). MMP9 (grey) signal associated with GFP+ hypertrophic chondrocytes (bottom panels). **i**. Electron micrographs with immunogold labelling showing strong LAMP1 (15nm gold particles; yellow arrowheads) in lysosome-rich (LYS) SCs with a large ER relative to vATPase-labelled (10nm gold; orange arrowheads) OCs. Supplementary figure 6d to h (n =4-5) and i (n=3) independent biological samples.

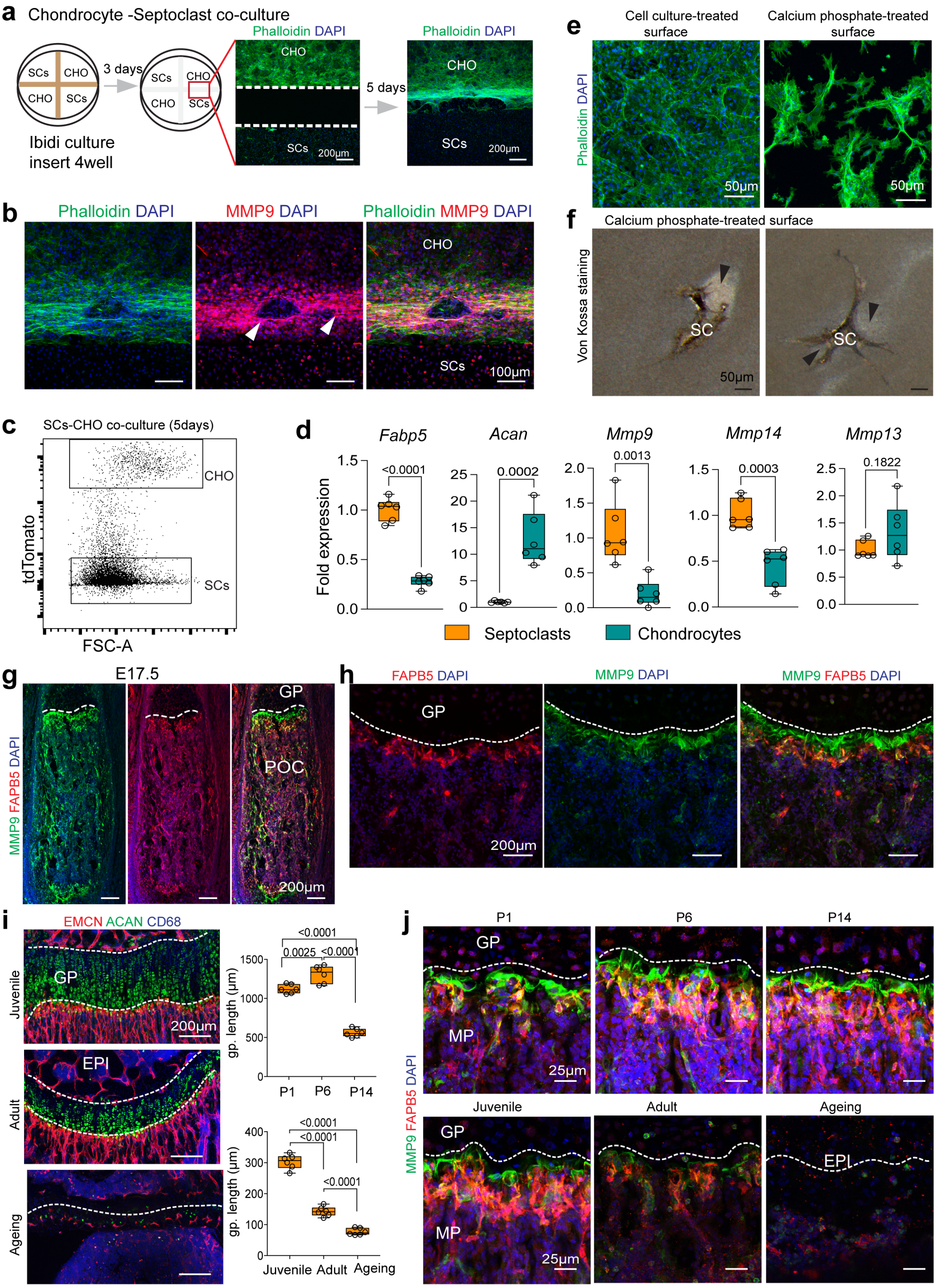

Supplementary Figure 7. Septoclasts MMP9 expression and resorption of cartilage.

a and **b**. Culture of septoclasts (SC) and chondrocytes (CHO) in 4-well insert micro dishes. Analysis of SC-CHO interactions 5 days after removal of insert (right image) (**a**). High

expression of MMP9 at the SC-CHO interface (red, arrowhead) (**b**). Phalloidin (green), DAPI (blue).**c** and **d**. FACS gating strategy for sorting of septoclasts (SCs, tdTomato-) and chondrocytes (tdTomato+) cells (c). RT-qPCR analysis shows that cell type specific marker gene *Fabp5*, *Acan,* and *Mmp9*, *Mmp14*, *Mmp13* expression in coculture septoclasts and chondrocytes (n=6 samples of three independent experiments; data are presented as mean values +/- SEM. Statistical analysis by Mann-Whitney test (two-tailed). Source data are provided in Source Data file. (d). **e** and **f**. Septoclasts on regular and calcium phosphate-treated culture substrate (osteo assay surface). Higher phalloidin staining and increased protrusion formation (**c**). Removal of calcium phosphate (arrowheads) by OCs (**d**). **g** and **h**. Tile scan confocal image of E17.5 long bone stained for FABP5 (red) and MMP9 (green). High expression of MMP9 decorates the edge of the primary ossification centre (POC) near growth plate cartilage. **i**. Representative confocal image showing changes in the growth plate (GP) during bone ageing. Chondrocytes (Acan, green), ECs (EMCN, red) and nuclei (DAPI, blue) labels. Graphs show alteration in length of growth plate during postnatal development and ageing (n=6 independent biological samples; data are presented as mean values +/- SEM, p-values, Statistical analysis performed using Tukey multiple comparison test (one-way Anova). Source data are provided in Source Data file. **j**. Representative confocal images of wild-type metaphysis at P1, P6 and P14. Note increase of FABP5+ (red) SCs and robust MMP9 immunosignal during postnatal development but profound decrease in adult and ageing samples (bottom row). Metaphysis (MP), growth plate (GP) and epiphyseal line in aged bone (EPI) are indicated. Supplementary figure 7a, b, e, f (n=4) independent in vitro experiments, and g, h, j (n =4) independent biological samples.

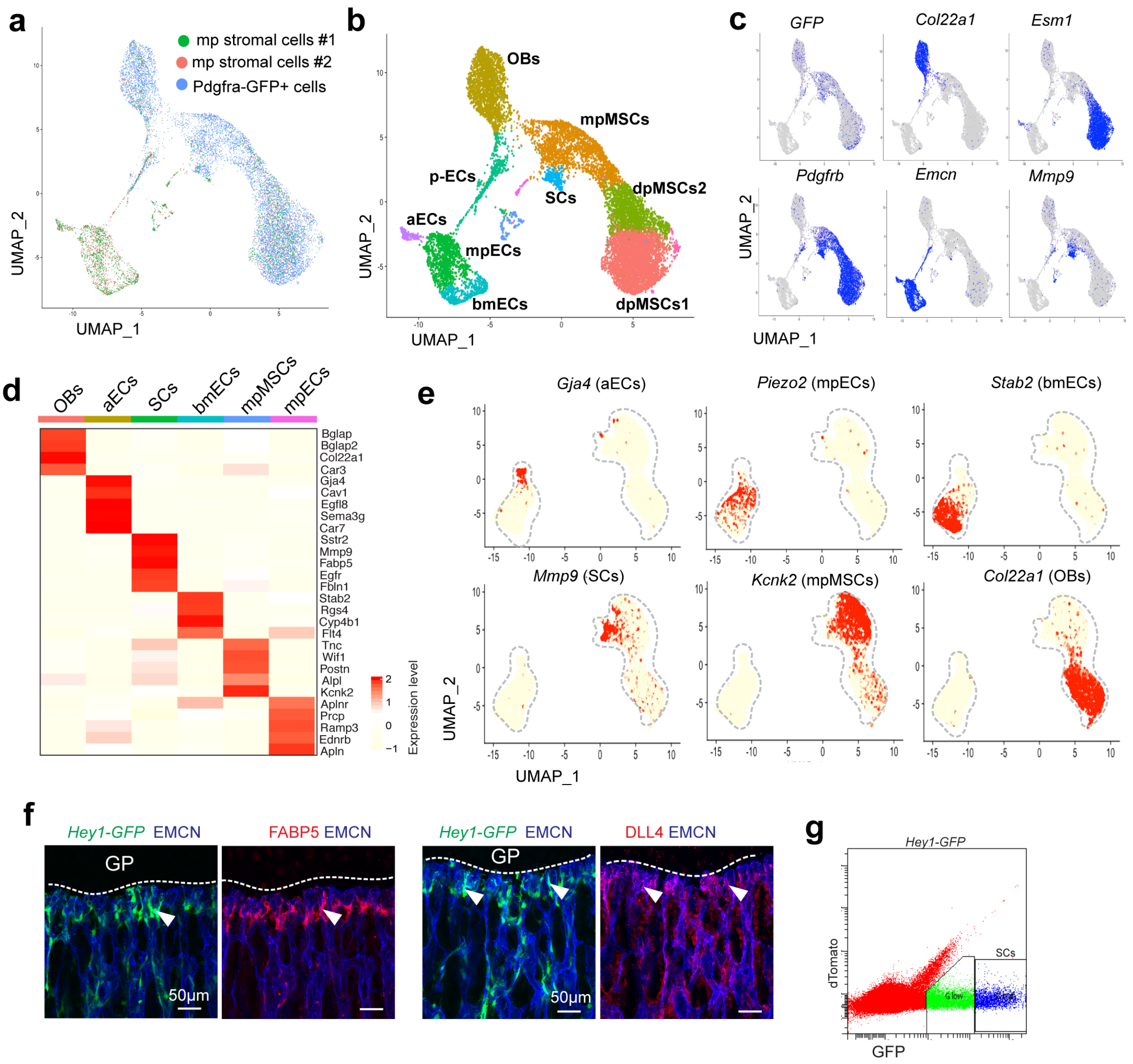

Supplementary Figure 8. Analysis of metaphyseal bone stromal cells.

a to **c**. UMAP plots of merged *Pdgfra-GFP+* and BMSC scRNA-seq data sets (**a**) and colour-coded clustering of cell populations (**b**) with expression of selected cell-type-specific marker genes (**c**). Osteoblast lineage cells (OBs), septoclasts (SCs), arterial ECs (aECs), bone marrow ECs (bmECs), metaphyseal ECs (mpECs), proliferating ECs (p-ECs), metaphyseal mesenchymal stromal cells (mpMSCs), and diaphyseal mesenchymal stromal cells (dpMSCs1, dpMSCs2). **d** and **e**. Heatmap with 5 top marker genes for OBs, SCs, aECs, bmECs, mpECs, and mpMSCs (**d**). Feature blots of population-specific markers: *Gja4* – aECs; *Piezo2* – mpEC; *Stab2* – bmEC; Mmp9 – SCs; Kcnk2 – mpMSCs; Col22a1- OBs (**e**). **f**. Representative confocal images of 3-week-old femur showing expression of *Hey1-GFP* (green) Notch reporter in FABP5+ (red) SCs (arrowheads). DLL4 (red) immunosignals in bud ECs (**f**). Growth plate

(GP) is indicated. **g**. Representative gating strategy for the FACS sorting of GFP^{high} cells from 3-week-old *Hey1-GFP* metaphysis for SCs isolation. Supplementary figure 8f (n =4) independent biological samples.

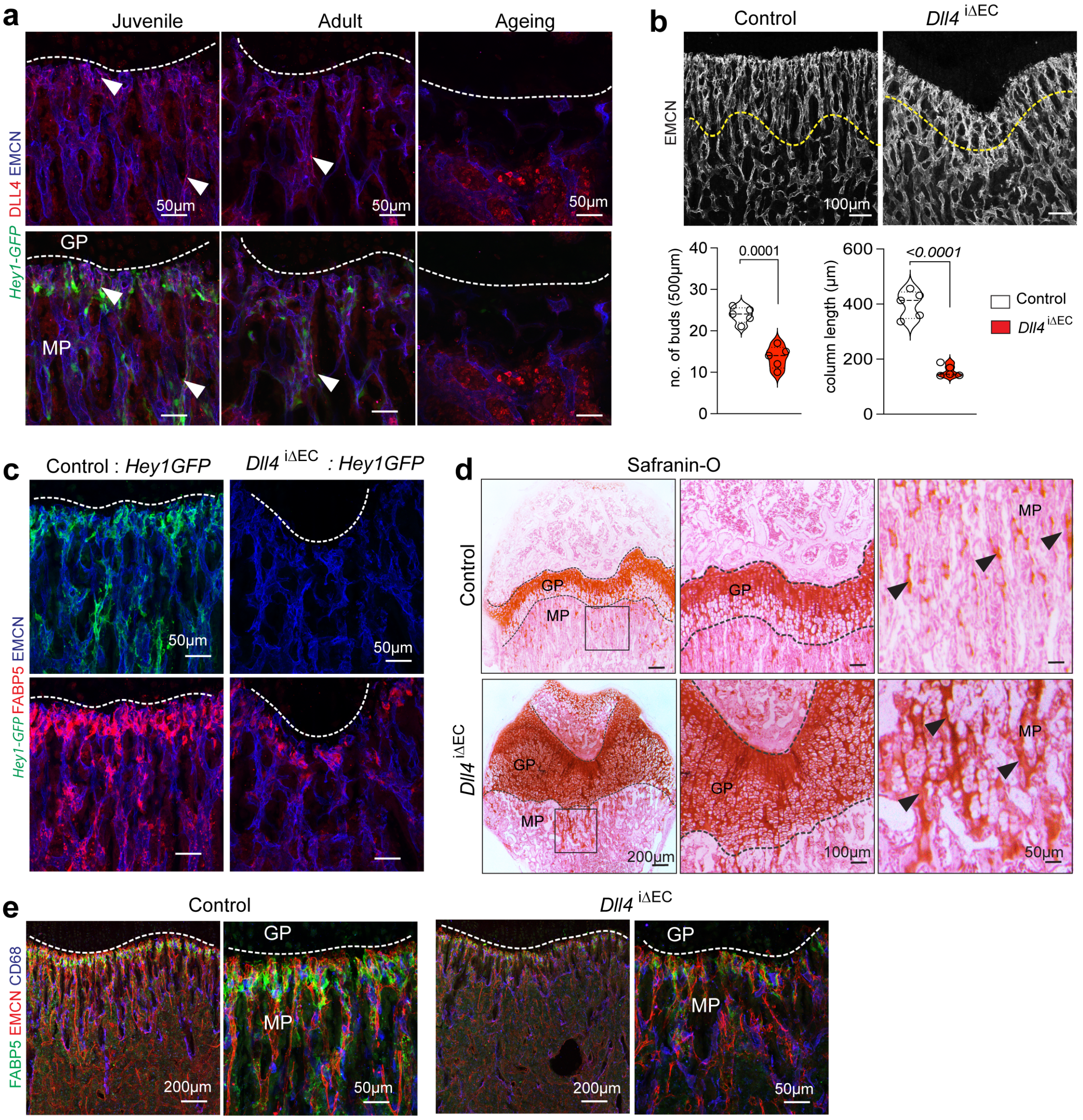

Supplementary Figure 9. Endothelial DLL4 activate Notch signalling in septoclasts.

a. DLL4 (red, arrowheads) immunostaining and *Hey1-GFP* reporter expression in femurs from different age groups. ECs, EMCN (blue). **b**. Representative confocal images showing metaphyseal bone vasculature (Emcn, gray) of 3-week-old *Dll4* $^{i\Delta EC}$ mutant and littermate control. Quantitation of vessel buds and column length in *Dll4* $^{i\Delta EC}$ mutants relative to controls. n=5 control and mutant bone; data are presented as mean values +/- SEM. Statistical analysis by Mann-Whitney test (two-tailed). Source data are provided in Source Data file. **c**. *Hey1-GFP* expression and FABP5 signals are reduced in *Dll4* $^{i\Delta EC}$ mutants. **d**. Safranin-O staining showing increase of growth plate area and cartilage remnants (arrowheads) in the *Dll4* $^{i\Delta EC}$ mutant metaphysis compared to control. Panels on the right show higher magnification of boxed area

on the left. **e**. Reduction of FABP5+ (green) but not CD68+ (blue) OCs in *Dll4* $^{i\Delta EC}$ mutant bone. ECs, EMCN (red). Supplementary figure 9a, c, d, e (n =5) independent biological samples.

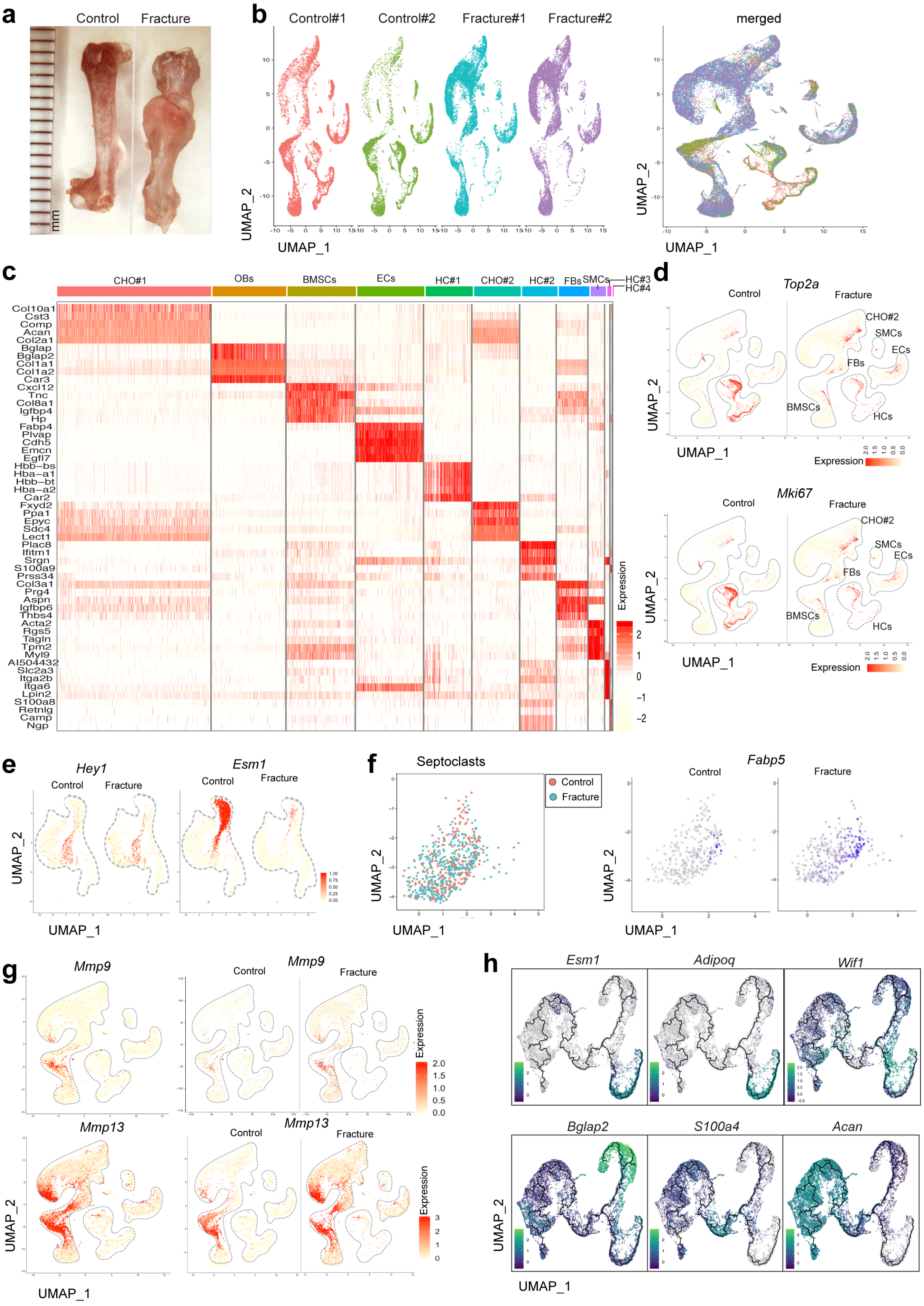

Supplementary Figure 10. scRNA-seq analysis of healing bone fractures. a. Freshly isolated 12-week-old control and PFD14 femurs. **b**. Independent control and PFD14 scRNA-

seq replicates displayed in individual and merged UMAP plots. **c** and **d.** Heatmap showing the top 5 marker genes for each cluster (**c**). Cell proliferation markers *Top2a* and *Mki67* are displayed in feature plots. Proliferating cells are increased in CHO#2, SMCs, ECs, FBs and BMSCs are increased in fracture compared to control scRNA-seq data (**d**). **e**. BMSC subcluster in UMAP plots shows reduction of *Esm1*+ dpMSCs but not of *Hey1*+ mpMSCs in PFD14 bone relative to control. **f**. Control and PFD14 SCs cluster together but *Fabp5* expression is increased after fracture. **g**. Fracture BMSC sc-RNA seq data shows elevated *Mmp9* and *Mmp13* expression in PFD14 samples.**h**. Monocle trajectory analysis of BMSC differentiation during callus formation. Expression of marker genes is displayed in trajectory.

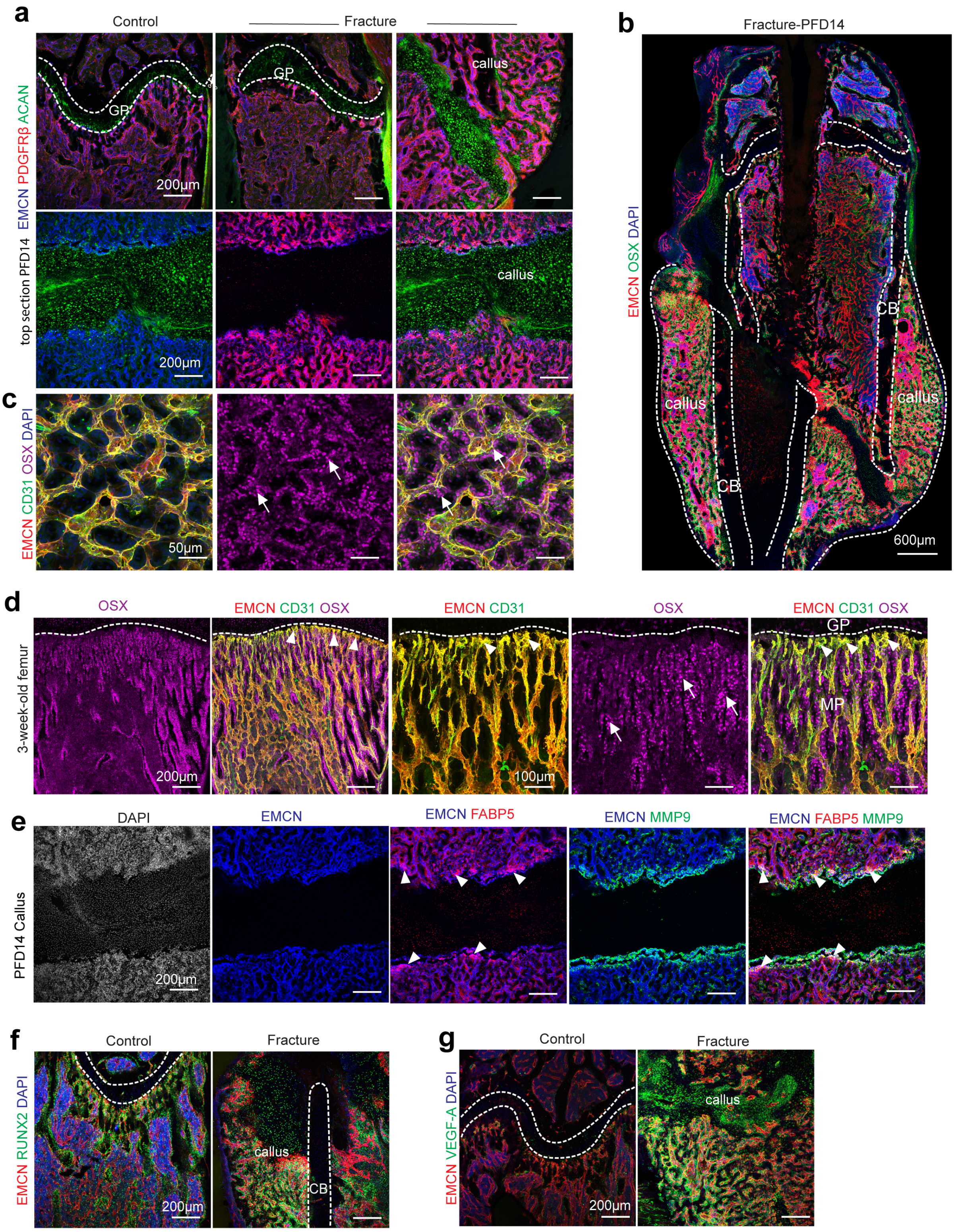

Supplementary Figure 11. Fracture repair and SC expansion. a. Confocal image showing control and PFD14 metaphysis area as well as the PFD14 callus. ECs (EMCN, blue), BMSCs (PDGFRβ, red) and callus (ACAN, green). **b** and **c**. Tile scan confocal image of PFD14 bone showing EMCN+ (blue) ECs and OSX+ (green) osteoprogenitor cells. Nuclei, DAPI (blue)

(**b**). High magnification images showing $CD31^{hi}$ (green) and $EMCN^{hi}$ (red) vessels (arrowheads) in proximity of callus chondrocytes. OSX+ (purple) cells (arrows) are abundant around vessels (**c**). **d**. Section of 3-week-old wild-type metaphysis showing $CD31^{high}$ (green) and $EMCN^{high}$ (red) distal vessel buds (arrowheads) in proximity of growth plate chondrocytes. OSX+ (purple) osteoprogenitors (arrows) are associated with vessel columns.**e**. Confocal images of PFD14 callus with FABP5+ (red) SCs (arrowheads), high MMP9 (green) signal at the leading edge of the callus vasculature (Emcn, blue). **f** and **g**. Representative confocal image of control metaphyseal region and PFD14 callus showing RUNX2+ cells (**f**) and increase of VEGFA signal (**g**) in PFD14 callus chondrogenic area relative to control metaphysis. Supplementary figure 11a to g (control =5 and fracture =4) independent biological samples.
