# Supplementary material for: Mesenchymal stromal cell-derived septoclasts resorb cartilage during developmental ossification and fracture healing
Source: Nat Commun. 2022 Jan 28;13:571. doi: 10.1038/s41467-022-28142-w (PMC8799643; doi:10.1038/s41467-022-28142-w)
Supplement: Supplementary file 3 — Reporting summary [file 41467_2022_28142_MOESM3_ESM.pdf]

nature portfolio

Corresponding author(s): Ralf H. Adams

Last updated by author(s): Dec 14, 2021

Reporting Summary

Nature Portfolio wishes to improve the reproducibility of the work that we publish. This form provides structure for consistency and transparency in reporting. For further information on Nature Portfolio policies, see our Editorial Policies and the Editorial Policy Checklist.

Statistics

For all statistical analyses, confirm that the following items are present in the figure legend, table legend, main text, or Methods section.

n/a	Confirmed	
☐	☒	The exact sample size (n) for each experimental group/condition, given as a discrete number and unit of measurement
☐	☒	A statement on whether measurements were taken from distinct samples or whether the same sample was measured repeatedly
☐	☒	The statistical test(s) used AND whether they are one- or two-sided<br>*Only common tests should be described solely by name; describe more complex techniques in the Methods section.*
☐	☒	A description of all covariates tested
☐	☒	A description of any assumptions or corrections, such as tests of normality and adjustment for multiple comparisons
☐	☒	A full description of the statistical parameters including central tendency (e.g. means) or other basic estimates (e.g. regression coefficient) AND variation (e.g. standard deviation) or associated estimates of uncertainty (e.g. confidence intervals)
☐	☒	For null hypothesis testing, the test statistic (e.g. F, t, r) with confidence intervals, effect sizes, degrees of freedom and P value noted<br>*Give P values as exact values whenever suitable.*
☒	☐	For Bayesian analysis, information on the choice of priors and Markov chain Monte Carlo settings
☒	☐	For hierarchical and complex designs, identification of the appropriate level for tests and full reporting of outcomes
☒	☐	Estimates of effect sizes (e.g. Cohen's d, Pearson's r), indicating how they were calculated

Our web collection on statistics for biologists contains articles on many of the points above.

Software and code

Policy information about availability of computer code

Data collection	RNA sequencing by NestSeq500, MiSeq (Illumina); Confocal immunofluorescence image were collected from leica TCS-SP8; Cell sorting and analyzed by FACS Aria II cell sorter (BD Bioscience); GraphPad Prism (version 7). Adobe Photoshop 2020 and Adobe illustrator 2020.
Data analysis	Data analysis and statistical tests were done using GraphPad Prism (Version 9). Confocal images were analyzed with Volocity 6.3 (Quorum Techonology) or imageJ(Version:2.0.0-re-69/1.52p).<br><br>Single cell RNA sequencing data analysis<br>Read Data pre-processing<br>Sequencing results were demultiplexed and converted to FASTQ format using Illumina ‘s bcl2fastq software. Raw reads were processed using fastp (version 0.20) excluding reads with an average quality score of less than 20, trimming the ends of reads base by base with a quality score less than 20 and requiring all reads to be their minimum expected length, based on the number of cycles during sequencing for the first read, containing the cellular barcode and UMI.<br><br>Read Mapping<br>Preprocessed read data was aligned to the mouse reference genome (mm10, Gencode M23 with H2B-GFP added) with STAR (version 2.7.3a) through its single-cell functionality STARsolo, using parameters to mirror unfiltered 10x Genomics CellRanger 3 output for sample demultiplexing, barcode processing and transcript counting. The official 10x Genomics whitelist for the respective Chromium version was used.<br><br>Count matrix Pre-processing<br>Datasets were analysed with default settings of the respective tools, if not specified otherwise. Preprocessing of the feature-count-matrix output by STARsolo was performed in R (version 3.6.0) using the scran (version 1.14.6), scater (version 1.14.6), SingleCellExperiment (version 1.8) and ggplot2 (version 3.3.2) packages. All visualisation was done using ggplot2 functions. Raw count matrices were loaded into

SingleCellExperiment objects for storage and manipulation. The EmptyDrops function from the DropletUtils package was used to pre-filter the feature-count-matrix. Per cell and per feature metrics were calculated with the appropriate functions of the scater package and used to exlude low-quality cells. Cells with less than 1500 (mpMSC) or 1000 (Pdgfra-GFP) features respectively as well as more than 15% transcripts of mitochondrial origin and 30% of ribosomal origin were filtered out. By these strict thresholds mostly remaining cells of the haematopoietic lineage and low quality cells were removed. Repeating the analysis without these cut-offs revealed no additional desired cell types. Only features present in more than 10 cells were retained. A total of 4,386 cells for the mpMSC datasets and 11,242 for the Pdgfra-GFP dataset were used for further Analysis. The mean numbers of detected genes per cell ranged from 2,120 in the Pdgfra-GFP dataset to 2,676 in the second replicate of the mpMSC dataset.

Clustering & Visualisation
Default settings were used, if not specified otherwise. Data normalization was performed using the normalize function provided by the scran package. The identification of highly variable genes, Cell cycle scoring, UMAP dimensionality reduction, Louvain clustering with multilevel refinement and the identification of marker genes were performed using Seurat (version 3.1.5). Always the top 5000 highly variable features were reported and used for principal component analysis (PCA) . All clustering steps were done using 500 starts and 100 iterations per start. Marker genes were calculated with Seurats FindAllMarkers function, using its implementation of the non-parametric Wilcoxon Rank sum test. Cell identities were manually annotated based on known marker genes.

Pdgfra-GFP Datasets
Variable genes were determined and then used for PCA. Cell cycle phases were classified and all genes were scaled and centered. For this scaling step only, the sum of mitochondrial reads per cell and the difference between the G2M and S phase scores as calculated earlier were regressed out. The first 30 principal components (PCs) were chosen and used for UMAP visualisation and clustering with multilevel refinement (resolution set to 0.6). Based on manually determined cell identities, the dataset was subset. Scaling, PCA, UMAP visualisation and Louvain clustering were rerun on the subset Pdgfra-GFPs dataset using the first 15 PCs with a resolution of 0.3. Filtering this way had to be repeated once more, with the same settings, to remove a small proliferative cell population that was not properly captured in the clustering steps before.
mpBSC Dataset
The procedure was highly similar to the analysis of the Pdgfra-GFPs dataset. To account for possible systematic technical effects between the replicates, the two pre-processed replicates were integrated using Seurat's alignment method for data integration (Butler et al 2018, Stuart et al, 2019) using the first 40 PCs. Briefly, this method uses canonical correlation analysis to learn the shared gene correlation structure across two datasets and then aligns them in lower-dimensional space thereby correcting for batch-effects. After integration, all genes were scaled and centered and the first 20 PCs were chosen for UMAP visualisation and Louvain clustering at a resolution of 0.4. Cell identities were determined as before and only cell identities of interest were retained. Scaling, PCA, UMAP visualisation and Louvain clustering were rerun, using the first 20 PCs and a clustering resolution of 0.4.

Merged Datasets
The pre-processed mpMSC and the final subset Pdgfra-GFP datasets were integrated using Seurat's integration method as before. Scaling, PCA, UMAP visualisation and Louvain clustering were run on the merged dataset using the first 25 PCs and a clustering resolution of 0.4. In this fashion also the following final clustering step was performed. After removing all cell identities not of interest for further Analysis, a dimensionality of 15 PC was used for UMAP visualisation and a resolution of 0.3 for Louvain clustering.

Trajectory analysis
Monocle was used for pseudotime trajectory analysis. We imported all information from Seurat objects to Monocle CDS objects and then performed dimensionality reduction using its DDRTree method with parameters max_components=2 and norm_method="vstExprs". The top 100 marker genes as determined earlier by log2 fold-change for each cluster were used as ordering genes for the trajectory. The resulting Trajectory was then plotted with default settings.

For manuscripts utilizing custom algorithms or software that are central to the research but not yet described in published literature, software must be made available to editors and reviewers. We strongly encourage code deposition in a community repository (e.g. GitHub). See the Nature Portfolio guidelines for submitting code & software for further information.

Data

Policy information about availability of data

All manuscripts must include a data availability statement. This statement should provide the following information, where applicable:
- Accession codes, unique identifiers, or web links for publicly available datasets
- A description of any restrictions on data availability
- For clinical datasets or third party data, please ensure that the statement adheres to our policy

The single cell RNA-sequencing datasets are available at Gene Expression Omnibus (GEO, https://www.ncbi.nlm.nih.gov/geo/). The accession number GSE154076 and GSE154247. The mouse reference genome (mm10, Gencode M23)

Field-specific reporting

Please select the one below that is the best fit for your research. If you are not sure, read the appropriate sections before making your selection.

☒ Life sciences ☐ Behavioural & social sciences ☐ Ecological, evolutionary & environmental sciences

For a reference copy of the document with all sections, see nature.com/documents/nr-reporting-summary-flat.pdf

Life sciences study design

All studies must disclose on these points even when the disclosure is negative.

Sample size	No specific statistical methods were used to predetermine sample size. Sample size were chosen based on previous experience. (ref. kusumbe et al. Nature 2014; Ramasamy et al., Nature 2014; Sivaraj et al., Elife 2020; Sivaraj et al., Cell Report 2021).
Data exclusions	Cells are excluded in the single cell RNA-seq data. Per cell and per feature metrics were calculated with the appropriate functions of the scater package and used to exlude low-quality cells. Cells with less than 1500 (mpMSC) or 1000 (Pdgfra-GFP) features respectively as well as more than 15% transcripts of mitochondrial origin and 30% of ribosomal origin were filtered out. By these strict thresholds mostly remaining cells of the haematopoietic lineage and low quality cells were removed. Repeating the analysis without these cut-offs revealed no additional desired cell types. Only features present in more than 10 cells were retained.
Replication	scRNA-seq experiments was performed duplicate and other all experiments were repeated at least three times and performed independently to ensure reproducibility. All the attempts of replication experiments were successful.
Randomization	No formal method of randomization was used. All experiments involving wildtype mice were performaed on inbred C57Bl6 strain with male mice of same age group. For mutant studies we used both male and female of same age group, and phenotype were always compared between same age and sex of animals. For fracture model we used age matched female mice.
Blinding	N/A

Reporting for specific materials, systems and methods

We require information from authors about some types of materials, experimental systems and methods used in many studies. Here, indicate whether each material, system or method listed is relevant to your study. If you are not sure if a list item applies to your research, read the appropriate section before selecting a response.

Materials & experimental systems

n/a	Involved in the study	
☐	☒	Antibodies
☒	☐	Eukaryotic cell lines
☒	☐	Palaeontology and archaeology
☐	☒	Animals and other organisms
☒	☐	Human research participants
☒	☐	Clinical data
☒	☐	Dual use research of concern

Methods

n/a	Involved in the study	
☒	☐	ChIP-seq
☐	☒	Flow cytometry
☒	☐	MRI-based neuroimaging

Antibodies

Antibodies used

All antibody details (clone and manufacturer) are included in methods and also listed below:
Antibody Catalog number Manufacturer Concentration
Endomucin sc-65495 Santa Cruz Biotechnology 1:100
CD31 AF3628 R&D 1:100
FABP5 C312991 Lifespan Bioscience 1:100
CD68(FA-11) ab53444 abcam 1:200
Pdgfrbeta AF1042 R&D 1:100
NG2 AB5320 Milipore 1:100
CD146 ab75769 Abcam 1:100
Osterix ab22552 abcam 1:300
GFP ab13970 abcam 1:200
MMP9 AF909 R&D 1:200
MMP13 18165-1-AP Proteintech 1:100
MMP14 PA5-13183 Invitrogen 1:100
ACAN AB1031 Milipore 1:100
LAMP1 553792 BD Pharmingen 1:100
vATPaseB1/B2 200839 R&D 1:100
Ki67 ab15580 Abcam 1:100
Dll4 AF1389 R&D 1:50
Runx2 ab192256 Abcam 1:200
Vegfa (EP1176Y) ab52917 Abcam 1:200
Phalloidin Alexa Fluor 488 A12379 Thermo Fischer Scientific 1:100
Alexa Fluor 488 A21208 Thermo Fischer Scientific 1:100

	Alexa Fluor 546 A11056 Thermo Fischer Scientific 1:100<br>Alexa Fluor 594 A21209 Thermo Fischer Scientific 1:100<br>Alexa Fluor 647 A31573 Thermo Fischer Scientific 1:100<br>Alexa Fluor 647 A21447 Thermo Fischer Scientific 1:100
Validation	All antibodies used in the study have been commercially available and previously used by our group (ref. kusumbe et al. Nature 2014; Ramasamy et al., Nature 2014; Sivaraj et al., Elife 2020; Sivaraj et al., Cell Report 2021). The complete information and all validation information for each Ab as well as previous publications that have used each Ab can be found on the manufacturer's website.

Animals and other organisms

Policy information about studies involving animals; ARRIVE guidelines recommended for reporting animal research

Laboratory animals	All animals used in this study are Mus musculus species, C57/BL6 background strain independent of genotype. All mice were maintained in pathogen free standard condition as mentioned in the animal guidelines.<br>Transgenic mice were generated from our laboratory, Cdh5(PAC)-CreERT2, Pdgfrb-Cre ERT2, Dll4 lox/lox mice; and follwoing transgenic mice PdgfraGFP, Rosa26-mTG, VavCre, Acan-CreERT2, Hey1GFP were purchased from Jackson Laboratory. 3-week old male and female trangenic mice were used for most of the experiments.<br><br>For mutant experiment , mice were bred to Cdh5(PAC)Cre -ERT2 to generate inducible mouse model. Cre negative were used as litter mate control. Most of the experiments were performed at 3 weeks.<br><br>Fracture healing experiment were performed on 10-week-old female mice and analyzed in 14days later.<br>For septoclast developmental and aging experiments bone were analyzed embryo E15.5, postnatal day1 (p1), P6, P14, and 3, 12, 70-76 weeks old male mice.
Wild animals	No wild animals were used in the study.
Field-collected samples	No field collected samples were used.
Ethics oversight	All animal experiments were performed according to the international guidelines and laws, approved by local animal ethical committee University of Muenster and Max Planck Institute for Molecular Biomedicine with premissions granted by the Landesamt fur Natur, Umwelt und Verbraucherschutz (LANUV) of North Rhine-Westphalia. Germany.

Note that full information on the approval of the study protocol must also be provided in the manuscript.

Flow Cytometry

Plots

Confirm that:

- [x] The axis labels state the marker and fluorochrome used (e.g. CD4-FITC).
- [x] The axis scales are clearly visible. Include numbers along axes only for bottom left plot of group (a 'group' is an analysis of identical markers).
- [x] All plots are contour plots with outliers or pseudocolor plots.
- [x] A numerical value for number of cells or percentage (with statistics) is provided.

Methodology

Sample preparation	Bone marrow isolation was performed using crushing methodology, followed by MACS beads and quadroMACS (LS columns) were used for lineage depletion or bone marrow stromal cells enrichment. 3-weeks-old PdgfraGFP male mice were used for isolating septoclasts. We used femur and tibia are seperated by surgical dissection and bone marrow were collected. Bones were dissected into small size and digested with Collagenase for 30 min at 37° C. Cells were washed with 1%BSA and filtered to obtained a single cell suspension. The detailed BM isolation protocol is included in the methods section.
Instrument	FACS Aria II cell sorter (BD Bioscience)
Software	FACSDiva (BD Bioscience) was used for sorting and analysis.
Cell population abundance	Sorted cell quality were analysed by confocal microscopy.
Gating strategy	Single viable cells were gated initally forward and side scatter. GFP + cells were sorted.

- [x] Tick this box to confirm that a figure exemplifying the gating strategy is provided in the Supplementary Information.
